# Effects of bipolar irreversible electroporation with different pulse durations in a prostate cancer mouse model

**DOI:** 10.1038/s41598-024-60413-y

**Published:** 2024-04-30

**Authors:** Song Hee Kim, Jeon Min Kang, Yubeen Park, Yunlim Kim, Bumjin Lim, Jung-Hoon Park

**Affiliations:** 1 Biomedical Engineering Research Center, Asan Medical Center, Asan Institute for Life Sciences, 88 Olympic-ro 43-Gil, Songpa-gu, Seoul, 05505 Republic of Korea; 2 Department of Gastroenterology, Asan Medical Center, University of Ulsan College of Medicine, 88 Olympic-ro 43-Gil, Songpa-gu, Seoul, 05505 Republic of Korea; 3 Departments of Urology, Asan Medical Center, University of Ulsan College of Medicine, 88 Olympic-ro 43-Gil, Songpa-gu, Seoul, 05505 Republic of Korea; 4 Department of Convergence Medicine, Asan Medical Center, University of Ulsan College of Medicine, 88, Olympic-ro 43-Gil, Songpa-gu, Seoul, 05505 Republic of Korea

**Keywords:** Urological cancer, Prostate, Oncology, Translational research

## Abstract

Irreversible electroporation (IRE) is a non-thermal ablation technique for local tumor treatment known to be influenced by pulse duration and voltage settings, affecting its efficacy. This study aims to investigate the effects of bipolar IRE with different pulse durations in a prostate cancer mouse model. The therapeutic effectiveness was assessed with in vitro cell experiments, in vivo tumor volume changes with magnetic resonance imaging, and gross and histological analysis in a mouse model. The tumor volume continuously decreased over time in all IRE-treated groups. The tumor volume changes, necroptosis (%), necrosis (%), the degree of TUNEL-positive cell expression, and ROS1-positive cell (%) in the long pulse duration-treated groups (300 μs) were significantly increased compared to the short pulse duration-treated groups (100 μs) (all *p* < 0.001). The bipolar IRE with a relatively long pulse duration at the same voltage significantly increased IRE-induced cell death in a prostate cancer mouse model.

## Introduction

As the rate of early prostate cancer diagnosis boosts rises due to increased prostate-specific antigen testing, a growing demand arises for treatment methods with good oncological and functional outcomes^1^. Established treatment options for prostate cancer range from active surveillance to radical therapy, including prostatectomy^2^. Unfortunately, these therapy methods often result in side effects including urinary incontinence (9.4–18.3%) and impotence (87.0–93.9%)^3–5^. Consequently, focal treatment options, including cryotherapy, high-intensity focused ultrasound, and irreversible electroporation (IRE), are emerging as preferred initial treatment methods for early prostate cancer. Image-guided IRE has been reported as a nonthermal ablative method based on the formation of nanoscale defects in cell membranes, leading to cell death^6–8^. All cells in the electric field that are formed during IRE are affected and go through apoptosis, but adjacent tissues such as blood vessels, urethra, and nerves are not significantly affected, making it easier to preserve the normal structures^9,10^.

Recently, the image-guided IRE technique has been applied for the treatment of prostate, pancreatic, and liver cancers, and its safety and efficacy have been documented^11,12^ Focal IRE in patients with localized prostate cancer showed promising results in both oncological and functional outcomes. The monopolar IRE with required multiple electrodes is used to locally ablate prostate tumor tissues using the high electric field strength of voltages ranging from 1000 to 3000 V and pulse durations ranging from 50 to 100 μs^13–15^. The monopolar IRE may pose potential hazards, such as cardiac arrhythmias and severe muscle contractions during IRE procedure^12,16^ Thus, research on alternative IRE protocols is actively conducted to overcome these limitations of monopolar IRE for local treatment of prostate cancer. Bipolar IRE is a relatively new variant of IRE that uses alternating positive and negative pulses and has been investigated in animal studies to verify its efficacy and safety compared with monopolar IRE^17^. However, high voltages are still applied to obtain a therapeutic electrical field strength and require muscle relaxants to prevent muscle contraction^15,18^. The mechanisms of cell death caused by a pulsed electric field have been shown to vary with electrical parameters^19–22^, and the pulse duration significantly influences the pore expansion process and ablation ranges^23,24^. Organ-specific and tumor-specific electrical parameter-response studies are lacking, and much remains unknown about the clinical possibilities to destroy malignant tissues with irregular geometries and heterogeneous properties. Herein, we hypothesize that IRE with relatively long pulse duration may enhance the ablation ranges even though the voltage was lowered for localized treatment of prostate cancer. A simple modified IRE parameter may increase the therapeutic effects of IRE while reducing electrical field strength-related complications during the IRE procedure. Therefore, the purpose of the study was to investigate the efficacy and safety of bipolar IRE with different pulse durations and voltages in a prostate cancer mouse model.

## Results and discussion

### Cytotoxicity and viability induced by bipolar IRE with different pulse durations

**Figure 1:**
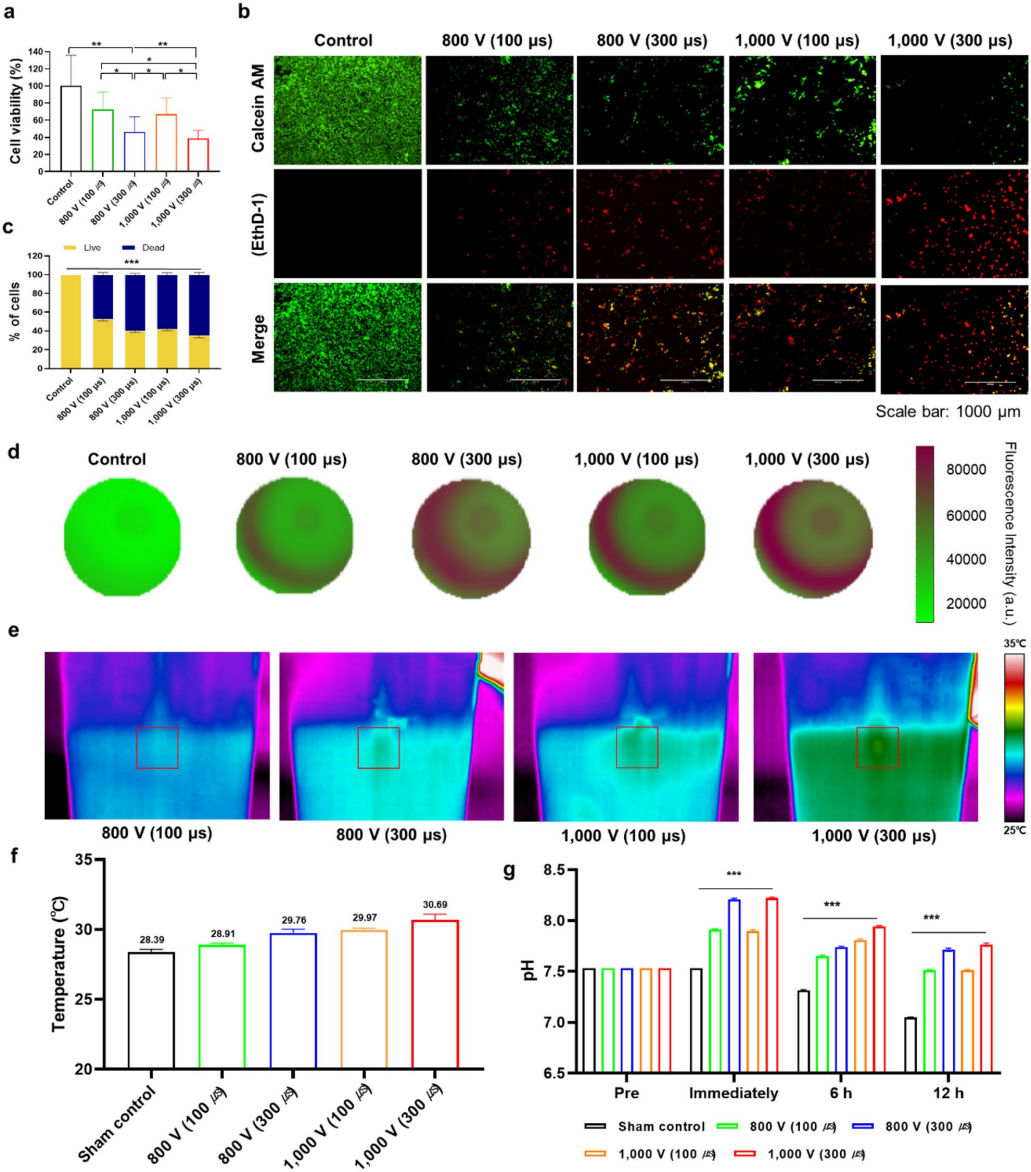
In vitro cellular experiments to evaluate various IRE parameters. (**a**) In vitro cytotoxicity of PC-3 cells using various voltages and pulse durations in the IRE treatment. (**b**) In vitro live and dead cell viability assay of PC-3 cells using various voltages and pulse durations in the IRE treatment. Live and dead cells were stained with Calcein AM (green color) and EthD-1 (red color), respectively (scale bar: 1000 μm). (**c**) Graph showing the quantitative results of the live and dead cell assay. **(d**) Representative microplate reader area-scanning images of intracellular ROS levels in the control and IRE-treated groups. (**e**) Representative thermal image obtained during IRE treatment. The red box indicates the region of interest (ROI). (**f**) Graph showing the temperature changes during IRE treatment. (**g**) Graph showing the pH changes after IRE treatment over time. Data are presented as the mean ± SD (*p < 0.05, **p < 0.01, ***p < 0.001).

The human prostate cancer cells (PC-3; American Type Culture Collection, Rockville, MD, USA) were cultured in vitro to perform IRE treatment under various parameters and confirm the viability of PC-3 cells by CCK-8 assay. The study by Miller et al. highlighted the fact that the cytotoxic effect of IRE is complex and varies depending on the pulse amplitude, length, number of pulses, and number of repeats^25^. Fig. 1a presents the correlation between the IRE parameter and cancer cell viability. Cell viability significantly decreased in all IRE-treated groups compared to the control group (all *p* < 0.05). Particularly, no significant difference was observed in cell viability between the 800 V (100 µs) and 1000 V (100 µs) groups (*p* = 0.537), although significant differences were observed in other comparisons (all *p* < 0.01). The cell viability in the 300 µs groups (800 V [43.32 ± 16.45] and 1000 V [38.56 ± 8.59]) was more efficient than that in the 100 µs groups (800 V [72.29 ± 18.50] and 1000 V [67.03 ± 16.39]) in achieving cell death. This significant reduction in cell viability indicates that the relatively long pulse duration had a pronounced effect on the death of PC-3 cells. In vitro cytotoxicity by IRE treatment was evaluated under various parameters through live and dead cell assays. As shown in Fig. 1b, as the pulse duration increased, live cells (Calcein AM, green color) decreased and dead cells (EthD-1, red color) increased. Quantification of the fluorescence signal of microscopic images (Fig. 1c) revealed that the death rate increased with the long pulse duration and high voltage (all *p* < 0.001). These results indicate that cancer cells can be effectively destroyed under IRE treatment with a pulse duration of 300 µs compared with that of 100 µs.

Reactive oxygen species (ROS) are highly reactive molecules that can react with various cellular biological components, including nucleic acids, proteins, organelles, and lipids^26^. The predominant cellular response following IRE treatment involves a rapid surge in ROS generation, which appears to be the principal trigger for the comprehensive disruption of intracellular homeostasis^27^. Consequently, one of the primary therapeutic functions of developed therapeutic modalities, such as photodynamic therapy, sonodynamic therapy, etc., including IRE, is to generate excessive cytotoxic ROS in the target tissue^26,28,29^. The levels of ROS production in PC-3 cells following the IRE treatment are shown in Fig. 1d. The ROS levels in the cell might arise from both the electrolysis and the release from mitochondrial leakage. The ROS signal intensity in the IRE-treated cells with a pulse duration of 300 µs was more prominent in comparison to those treated with a pulse duration of 100 µs. These qualitative observations imply that a pulse duration of 300 µs might exert a potential influence on cellular ROS generation.

### Temperature and pH changes by IRE parameters

Representative thermal images with maximum temperature during the IRE procedure are shown in Fig. 1e. The mean maximum temperatures in the IRE-treated groups were significantly higher than that in the sham control group. The highest temperature was 30.69 ℃ in the IRE-treated group (1000 V, 300 µs), however; the difference between the sham control and the IRE-treated (1000 V, 300 µs) groups was only 2.30 °C. IRE involves two distinct aspects: a biophysical, cellular-level response to repeated electric field exposure, and its therapeutic application to destroy targeted tissue volumes. When used properly, the non-thermal cell death mechanism in IRE therapy can effectively eliminate significant thermal damage surrounding tissues^30^. Although a slight temperature increase was observed, our data validated the feasibility of effective IRE ablation without thermal effects.

The pH level immediately after IRE treatment temporarily increased due to an oxidation reaction caused by the diffusion and migration of hydrogen or hydroxyl ions^26,31,32^. In the sham control group, pH levels were gradually decreased over time (Fig. 1g). In the IRE-treated group, pH levers were rapidly increased immediately after IRE treatment. The pH level in the IRE-treated group with 300 μs (800 V [8.21 ± 0.01] and 1000 V [8.22 ± 0.01]) was higher than that in the IRE-treated group with 100 μs (800 V [7.91 ± 0.01] and 1000 V [7.90 ± 0.01]) immediately after IRE treatment. However, pH levels were gradually decreased at the 6 h (800 V, 100 μs [7.65 ± 0.01], 800 V, 300 μs [7.74 ± 0.01], 1000 V, 100 μs [7.81 ± 0.01], and 1000 V, 300 μs [7.94 ± 0.01]) and 12 h (800 V, 100 μs [7.51 ± 0.01], 800 V, 300 μs [7.71 ± 0.02], 1000 V, 100 μs [7.51 ± 0.01], and 1000 V, 300 μs [7.76 ± 0.02]) after IRE treatment. Our findings support that increased applied voltage or pulse duration during the IRE procedure can promote cell death and increase necrotic area.

### In vivo tumor growth suppressed by IRE treatment in a mice tumor model

**Figure 2:**
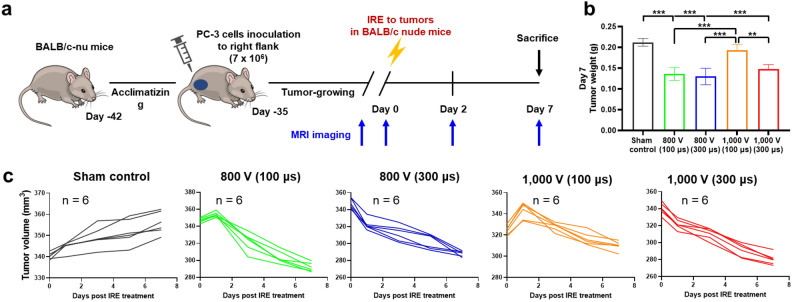
In vivo tumor suppression by IRE treatment in the PC-3 tumor model. (**a**) Schematic representation of the experimental design. (**b**) Extracted tumor weight on day 7 after IRE treatment. (**c**) Individual tumor volume change curves following each group, with the time synchronized to IRE treatment on day 0. Data are presented as the mean ± SD (n = 6, **p < 0.01, ***p < 0.001).

PC-3 cells were inoculated into BALB/c nude mice to establish a tumor model to investigate the efficacy of the various IRE parameters. The evaluation focused on whether an increased pulse duration was employed to facilitate regression of tumor growth in the PC-3 model. The detailed animal experiment schedule is indicated in Fig. 2a. Bipolar IRE treatment was conducted when the tumor volume reached 250 mm^3^–300 mm^3^. The results of tumor inoculation and growth curve analysis on PC-3 cells are shown in Supplementary Fig. 2a and b. After tumor inoculation, linear body weight and tumor volume changes were observed and gradually increased over time.

All IRE procedures were technically successful in all enrolled mice. The bipolar IRE-treated mice survived until the end of the study. Mild muscle contractions were observed in all mice during the IRE procedure. A limitation of the study was the inability to quantitatively evaluate the correlation between electric field strength and muscle contraction and arrhythmia. In comparison to bipolar IRE with long pulse duration-treated tumors, short pulse duration-treated tumors showed a brief delay of growth kinetics immediately after IRE treatment, although associated edema and swelling by IRE treatment masked temporary differences in tumor lesion size (Fig. 2). Recently, focal therapy has emerged as a viable alternative treatment option for small, localized prostate tumors^33,34^. We investigated the effects of different voltages and pulse durations, but we fixed the number of pulses (40). Although additional studies are needed to find optimal IRE parameters, IRE parameters with simple changes can effectively reduce tumor volume and minimize IRE-related complications.

### Follow-up magnetic resonance imaging findings

**Figure 3:**
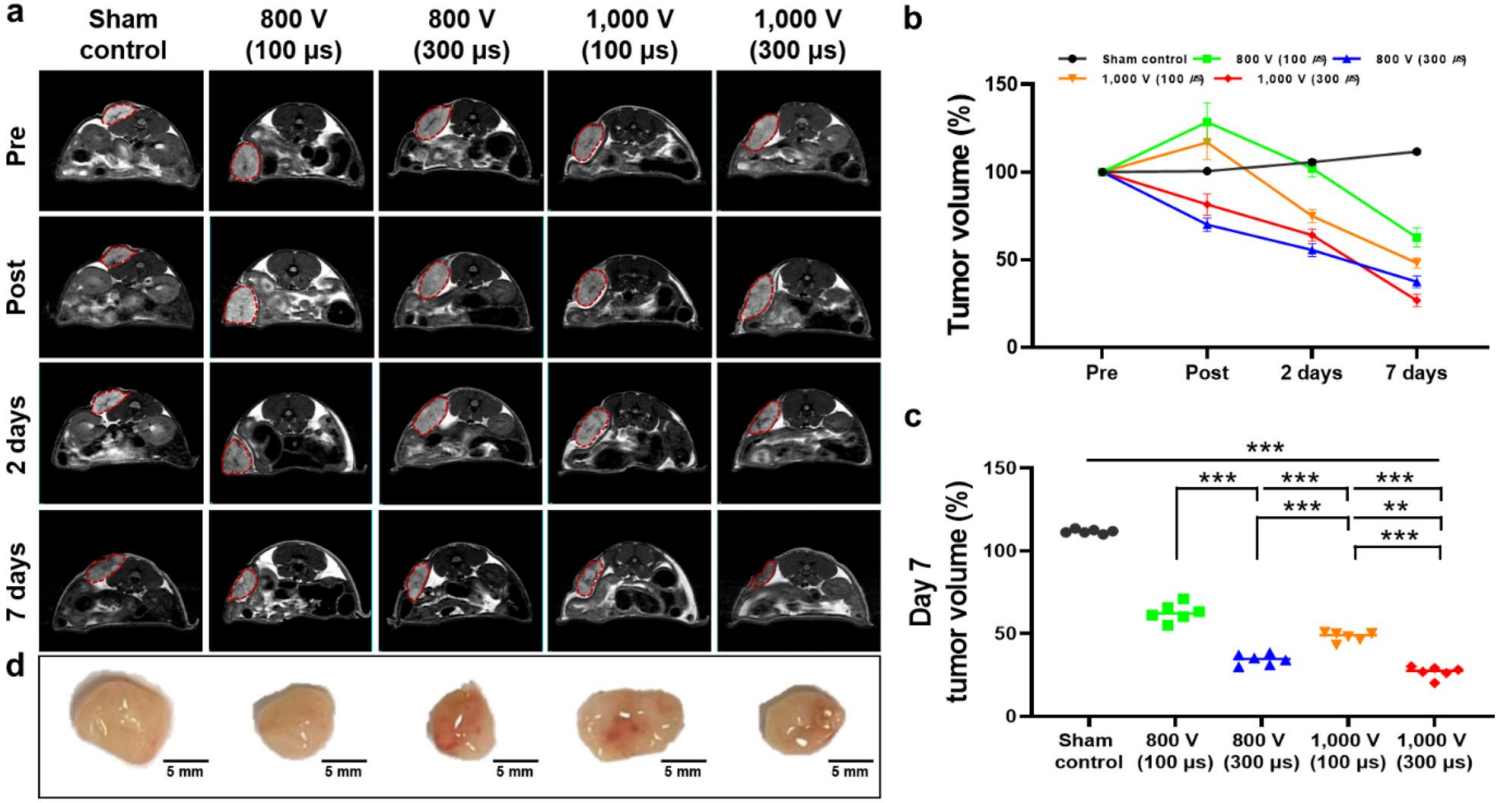
Magnetic resonance (MR) and gross findings. (**a**) Representative axial T_2_-weighted MR images of a PC-3 tumor mouse from each group over time. (**b**) The graph shows the percentage of tumor volume change curves after IRE treatment in all groups. (**c**) Individual tumor volume on day 7 from each group in (**b**). (**d**) Representative images of the extracted tumor on day 7 after IRE treatment. Data are presented as the mean ± SD (n = 6, **p < 0.01, ***p < 0.001).

Representative magnetic resonance (MR) images and the percentages of tumor volume are shown in Fig. 3. T_2_W images are useful for monitoring tumor changes^35^. One of the main reasons for this phenomenon would be that the cell density of the prostate carcinoma in vivo would be higher than that of other normal solid organs such as the prostate^36^. The tumor volume in the control group gradually increased over time. The IRE with 100 μs-treated groups showed a transient increase in tumor volume immediately after IRE treatment but a sustained decrease until day 7. A transient increase in tumor volume is associated with edema. Edema is observed in the treated point, which is a known feature of ablation by electroporation^37^. Especially, low pulse durations increase the osmotic pressure within cells, resulting in cell bulkiness^38^. In contrast, the tumor volume in the IRE with 300 μs groups continuously decreased over time. It suggests that increasing the pulse duration may induce more effective apoptosis or cell death than the groups with a short pulse duration. Meanwhile, the percentage of tumor volume was significantly lower in the 800 V (300 μs) group (34.33 ± 3.30%) and the 1000 V (300 μs) group (26.79 ± 3.54%) than in the 1000 V (100 μs) group (48.13 ± 2.95%, all *p* < 0.001). Increasing the voltage can strongly reduce the volume of the tumor but may have side effects such as severe muscle contraction or cardiac arrhythmia due to the formation of an excessive electric field^39^. Therefore, efficient electrical energy should be applied at a low voltage for effective electroporation^40^. IRE with relative long pulse duration could significantly extend had the ablation range and reduce electric field strength with a lowered voltage^24,30^. Our results demonstrated the volume of ablated tumor was significantly decreased with low voltage (800 V, 300 μs) compared to the low pulse duration with high voltage (1000 V, 100 μs). Our findings support that sufficient electroporation with a low voltage by applying a relatively long pulse duration was possible in a prostate cancer mouse model.

### Gross and histological findings

All mice were euthanized for gross and histological examinations, and the whole tumor tissue was successfully extracted and photographed with a digital camera (Fig. 3d). All IRE-treated tumor tissues were smaller than the sham control, no gross abnormalities caused by bipolar IRE treatment were observed.

**Figure 4:**
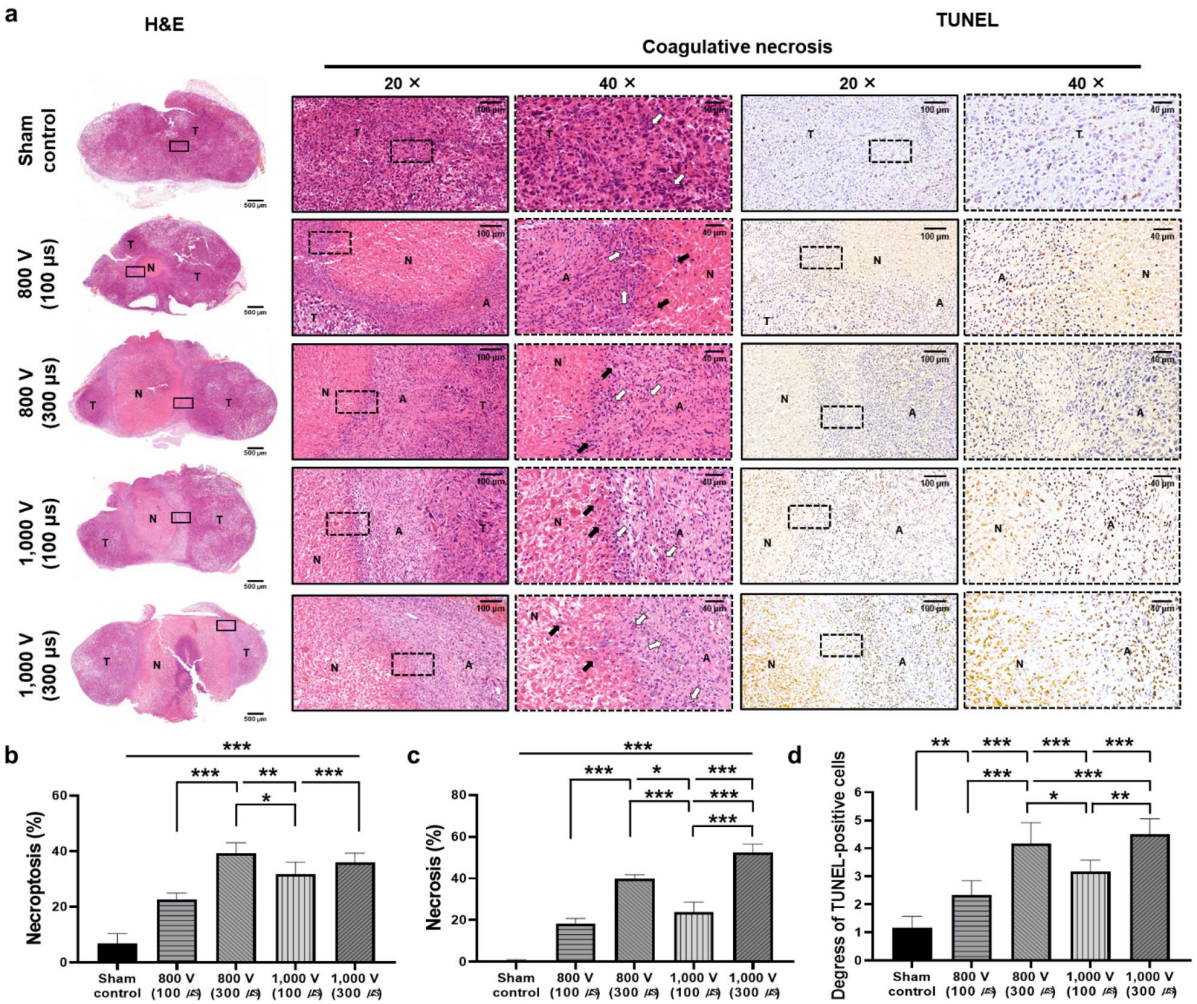
Representative microscopic images of tumor tissue at various IRE parameters. (**a**) Hematoxylin and eosin (H&E) staining and terminal deoxynucleotidyl transferase-mediated dUTP nick end labeling (TUNEL) assay. Images were taken under × 2, × 20, and × 40 magnifications for each field. The necrotic morphologies were indicated by the following symbols: black arrow, karyolysis; white arrow, karyopyknosis. Relative quantification of (**b**) necroptosis, (**c**) necrosis, and (**d**) TUNEL-positive cells in PC-3 tumors for each group. Data are presented as the mean ± SD (n = 6, *p < 0.05, **p < 0.01, ***p < 0.001).

Histologic findings with statistical significance are summarized in Supplementary Tables 1 and 2, and examples are shown in Fig. 4. The mean percentages of necroptosis and necrosis and the degree of terminal deoxynucleotidyl transferase-mediated dUTP nick end labeling (TUNEL)-positive deposition were significantly different between the study groups (all *p* < 0.001). The percentages of necroptosis and necrosis in the IRE-treated groups with 300 μs were significantly higher than in the IRE-treated groups with 100 μs. The PC-3 cells in tumor tissues are mainly distributed in a clustered pattern with high density and less intercellular substance. The IRE-treated groups showed significant differences in histological changes in the electrode regions near the epidermis. A homogeneous red-stained patch was observed in the electrode region, with a blurred cell structure and coagulative necrosis. In comparison with the sham control group, these clusters exhibited clear necrotic morphologies, such as karyolysis and karyopyknosis. Moreover, in the IRE-treated groups with long pulse durations, the necrosis border was well-defined, and a large area of necrosis was observed, accompanied by karyolysis and karyopyknosis. IRE with high voltage induces cell necrosis, followed by cellular and tissue debris, which triggers local or systemic inflammation and immune responses. Moreover, huge necrosis may induce severe bleeding and infection in the IRE-treated tumoral tissues, which are not suitable for anticancer therapy^9,41^. Our histological findings demonstrated that the highest necrosis area was shown in the 1000 V with 300 μs group, whereas the highest necroptosis area was observed in the 800 V with 100 μs group. These results demonstrate that bipolar IRE with different voltages and pulse durations significantly affects cellular necrosis. Although three observers conducted blinded analyses to evaluate interobserver variability, the histological analysis was subjectively determined according to the distribution and density of stained cells.

As demonstrated in other research, apoptosis is closely associated with both tumorigenesis and tumor regression^42,43^. Quantitatively assessing TUNEL-positive cell expression, the effects of various parameters on cell death within tumor tissues were investigated. The mean degrees of TUNEL-positive cell deposition were significantly different between the groups (*p* < 0.001). The TUNEL-positive cells in the IRE with 300 μs treated groups were significantly increased compared with the IRE with 100 μs treated groups (all *p* < 0.01). Despite the higher electric field strength of 1000 V with 300 μs, the highest TUNEL staining was observed at 800 V with 300 μs. No significant difference in the TUNEL-positive cell expression was observed between the 800 V and 1000 V groups at the same pulse duration. These results suggest that increasing pulse duration seems to be more efficient for tumor necrosis than increasing voltage. While the effects of IRE may vary depending on tissue and cellular diversity, these results may aid optimizing IRE based cancer treatment by increasing pulse duration.

### Immunofluorescence findings

**Figure 5:**
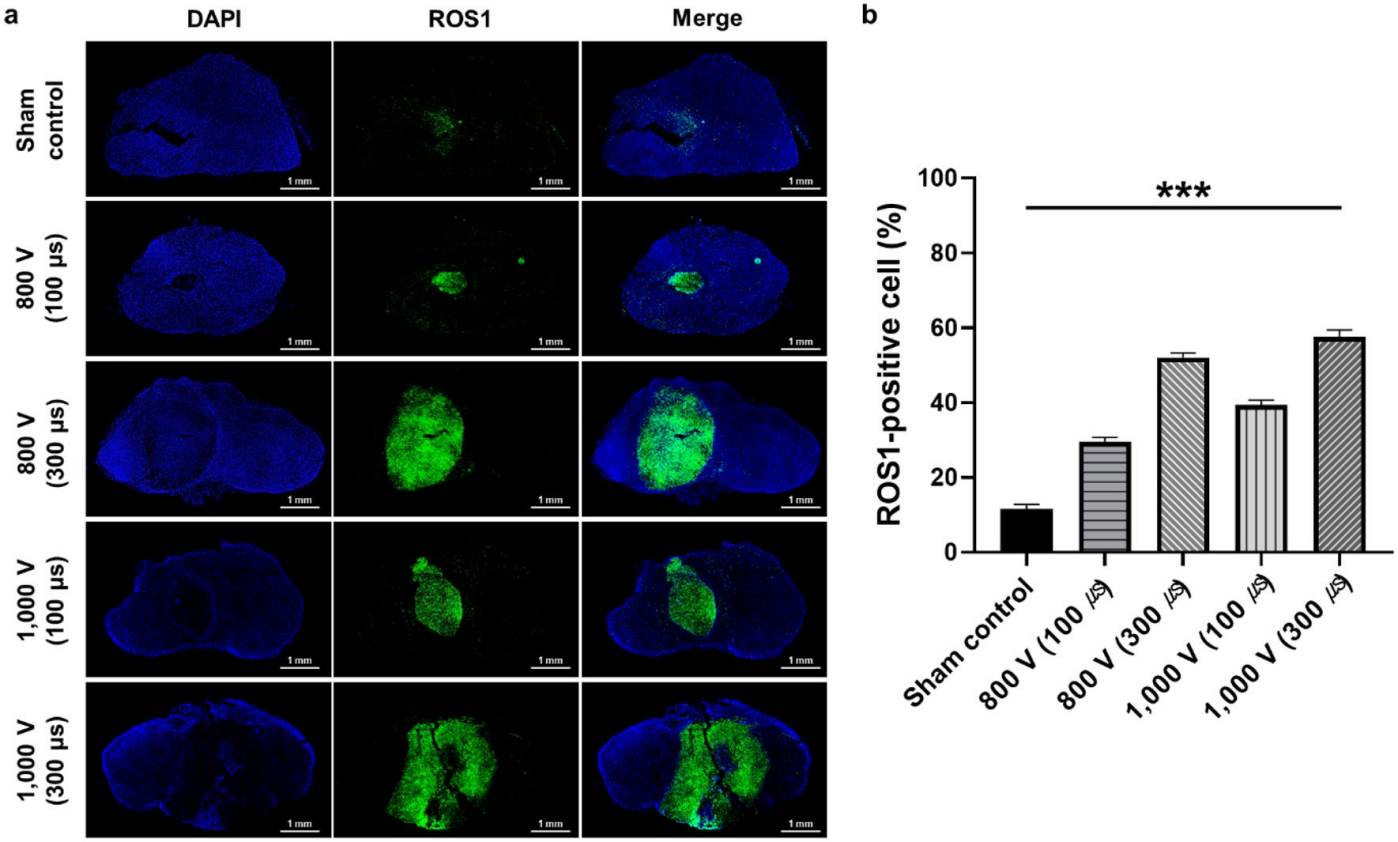
Immunofluorescence findings of tumor tissue at various IRE parameters and qualitative analysis of ROS1 expression. (**a**) Representative immunofluorescence (DAPI, blue, and ROS1, green) images of ROS1-positive cells in tumors tissue after IRE treatment. (**b**) Relative quantification of ROS1-positive cells in PC-3 tumor for each group. Data are presented as the mean ± SD (n = 6, ***p < 0.001).

ROS1 is a tyrosine kinase that functions as a growth and differentiation factor receptor^44^. Protein kinases are enzymes that play a key role in signal transduction within eukaryotic cells, governing various cellular processes, including metabolism, transcription, cell cycle, cytoskeletal dynamics, apoptosis, and differentiation, through the transfer of phosphate groups^45^. Qualitative analyses of ROS1 expression are presented in Fig. 5. Consistent with histologic findings, the bipolar IRE treatment significantly influenced the increase of ROS1-positive cell depositions in all groups (*p* < 0.001). Furthermore, IRE treatment with higher voltage and a longer pulse duration significantly increased the percentage of ROS1-positive cells, as indicated by the green fluorescence. This finding indicates a significant correlation between the high proportion of ROS1-positive cells and the areas of cellular damage within the tumor tissue. Although ROS1-positive cells, antibodies, and other immune mechanisms are yet to be explored, ROS1 expression may contribute to the significant marker for IRE-induced cell death in a prostate cancer mouse model.

## Conclusion

Based on the present study findings, this study successfully demonstrated the effects of bipolar IRE with different applied pulse durations in a prostate cancer mouse model. Bipolar IRE with a relatively long pulse duration at the same voltage significantly increased IRE-induced cell death in a prostate cancer mouse model. Considering the wide interval between the applied voltages and pulse duration, further studies are required to find the minimum required applied voltages with optimized pulse duration to enhance tumor necrosis and minimize IRE-related complications for patients with prostate cancer.

## Methods

### Cell culture

PC-3 (American Type Culture Collection, Rockville, MD, USA) cell was cultured in RPMI1640 (R8758; Sigma Aldrich, St. Louis, MO, USA) medium, containing 2 g/L glucose, 0.3 g/L L-glutamine, and sodium bicarbonate. The medium was supplemented with 10% fetal bovine serum (SH30919.03; GE Healthcare Life Sciences, Logan, UT, USA) and 1% penicillin–streptomycin (17-745E; Lonza Bioscience, Walkersville, MD, USA). All cells were cultured at 37 °C in a humidified environment containing 5% CO_2_ and sub-cultured with 0.025% trypsin–EDTA (Life Technologies, Gaithersburg, MD, USA).

### Bipolar IRE treatment in prostate cancer cells

An in vitro bipolar IRE treatment was performed on the PC-3 cells using a 2-needle array electrode made by stainless steel (BTX Harvard Apparatus, Holliston, MA, USA) with an electric pulse generator (ECM 830; BTX Harvard Apparatus). The distance between the two electrodes was 5 mm, and the length of each electrode was 10 mm (Supplementary Fig. 1a). Four parameters were evaluated at different pulse durations and voltages with a specified number of pulses (voltage: 800 V and 1000 V, pulse duration: 100 µs and 300 µs, number of pulses: 40, interval time of pulses: 0.1 s) (Supplementary Fig. 1b-d). In brief, the PC-3 cells (1 × 10^6^ cells/well) were seeded in 24-well plates and then electroporated at room temperature by direct contact with the electrode.

### Determination of cell cytotoxicity and cell viability

The in vitro cytotoxic effects of bipolar IRE treatment on PC-3 cells were determined using a CCK-8 assay. Cells were seeded in 24-well plates at a density of 1 × 10^6^ cells/well, and then IRE was performed after 12 h. After 24 h, the cells were washed with phosphate buffered saline (PBS) and added to a 10% CCK-8 solution (CCK-3000; DOJINDO, Kumamoto, Japan). After incubation for 3 h, the absorbance was measured at 450 nm using a microplate reader (Synergy HTX; BioTek, Winooski, VT, USA) and analyzed with Gen5 software (Agilent Technologies, Santa Clara, CA, USA). Percentages of live cells were calculated for the IRE-treated group compared to the control group for cell viability using the following formula: 100 × (Mean absorbance of the treatment group/Mean absorbance of the control group).

To perform the LIVE/DEAD assay with various IRE parameters, PC-3 cells (1 × 10^6^ cells/well) were seeded in 24-well plates and underwent IRE treatment. After IRE, the cells were incubated at room temperature with a mixture of Calcein AM and EthD-1 (L3224; Invitrogen, Carlsbad, CA, USA) for 30 min. The live cells were stained with Calcein AM, whereas the dead cells were stained with Ethd-1. After 30 min, the cells were washed three times with PBS and fixed with 4% paraformaldehyde (PFA) for 15 min. The stained cells were imaged using a fluorescence microscope (EVOS FL Auto; Thermo Fisher Scientific, Waltham, MA, USA), and ImageJ 1.53c software (National Institutes of Health, Bethesda, MD, USA) was used to analyze the fluorescence imaging.

### Temperature monitoring and pH measurement

The temperature changes and thermal images during IRE procedure on PC-3 cells in according to different voltage and pulse duration, as follows: (1) sham control; (2) 800 V and 100 µs; (3) 800 V and 300 µs; (4) 1000 V and 100 µs; (5) 1000 V and 300 µs were examined using a thermal camera (FLIR A400; Teledyne FLIR, Wilsonville, OR, United States). The temperature data with thermal image were stored as electronic files every 1 s during the IRE procedure. The maximum temperature was recorded in each experiment. A thermal camera was positioned from a 180-degree angle. All in vitro studies were repeated six times to maintain statistical reproducibility.

In vitro pH measurements were conducted after the IRE procedure using a pH meter (Orion Star^™^ A211 pH Benchtop Meter, Thermo Scientific, Waltham, MA, USA) at four different time points: before IRE, immediately after IRE, 6 h and 12 h after IRE. The pH meter was calibrated with standard buffer solutions. For each sample, a volume of 1 ml of the cell medium was collected and transferred to a clean container. The pH of the cell medium was then directly measured using the calibrated pH meter, and the average of triplicate measurements was recorded.

### Cell reactive oxygen species analysis

ROS levels in PC-3 cells were evaluated using 2',7'-dichlorofluorescin diacetate (DCFDA; ab113851; Abcam, Cambridge, UK), a cell-permeable fluorogenic dye. PC-3 cells (1 × 10^6^ cells/ml) were cultured in 24-well plates for 12 h, followed by cell IRE treatment under various parameters. After 24 h, the PC-3 cell culture solution was discarded and washed with PBS three times, then the cells were stained with a 5 µM DCFDA solution and incubated for 50 min at 37 °C. After staining, the staining buffer was removed; the cells were washed three times with PBS and fixed with 4% PFA for 15 min. At 485 nm excitation and 530 nm emission, the range of the relative fluorescence unit (RFU) was observed using the area scan mode of a microplate reader.

### Establishment of a tumor model in nude mice

This study was approved by the Institutional Animal Care and Use Committee of Asan Institute for Life Sciences (#2020-13-349) and conformed to US National Institutes of Health guidelines for handling laboratory animals. The study was carried out in compliance with ARRLVE guidelines. A total of 30 male BALB/c nude mice (5 week-old, JA BIO, Suwon, Korea) were used for the animal experiment after stabilization for 1 week. Mice were housed in a breeding environment that maintained a 12 h night and day cycle at a temperature of 20–24 °C and humidity of 44.5–51.8%.

To establish a mouse tumor model, subcutaneous inoculation was performed by injecting 7.0 × 10^6^ cells in a 200 µl volume of PBS under the right flank skin, using a 26 ½ G syringe, to allow tumors to form. The tumor was measured every 2–3 days using a caliper. Tumor volume was calculated by measuring the longest and shortest dimension and multiplying to provide a two-dimensional representation of the tumor area. Tumor volumes were calculated with the following formula: $V=({W}^{2}\times L)/2$, where W is the tumor width and L is the tumor length for BALB/c mice^46^. When the average volume of tumors reached 250 mm^3^, the mice were enrolled in the study. Animals were sacrificed when tumor volumes reached a maximum of 2000 mm^3^ or weight loss exceeded 15%^47^.

### IRE treatment in a nude mice tumor model

A total of 30 of 45 mice in which the tumor volume reached 250 mm^3^ were chosen. The remaining 15 mice were excluded from the study as they did not meet the tumor volume criteria, ensuring the reliability of the experimental outcomes. The enrolled mice were equally distributed into five groups, with six in each, according to the voltage and pulse duration, as follows: (1) sham control; (2) 800 V and 100 µs; (3) 800 V and 300 µs; (4) 1000 V and 100 µs; (5) 1000 V and 300 µs. A bipolar IRE procedure with a fixed number of 40 pulses was performed at different voltages and pulse durations in enrolled mice. The interval time between each bipolar pulse was set to 0.1 s, with both positive and negative pulse durations set to the same duration (Supplementary Fig. 1b-e). Mice were gas-anesthetized with 2% of isoflurane (Ifran^®^; Hana Pharm. Co., Seoul, Korea) and were kept under anesthesia during the procedure. A 2-needle array was inserted entirely into the center of the tumor and then electroporated. All mice were sacrificed 1 week after IRE treatment by inhalation of pure carbon dioxide.

### Magnetic resonance imaging monitoring

Magnetic resonance imaging (MRI) was performed to analyze the changes in tumor shape and volume after IRE treatment using a 9.4 T animal MRI system (Agilent Technologies, Santa Clara, CA, USA). The animals were anesthetized in an induction chamber of 5% isoflurane with 1:1 oxygen (2 L/min). Mice were placed on the animal bed with a mask for the maintenance dose of the anesthesia (2% isoflurane). T_2_-weighted (T_2_W) MRI images were obtained in the axial section of the tumors before the procedure and immediately, 2, and 7 days after IRE treatment. A T_2_W imaging sequence with a field of view (FOV) = 30.0 × 30.0 mm, matrix size = 256 × 256, slice thickness = 2 mm, repetition time (TR) = 4000 mS, echo time (TE) = 34 mS, and flip angle (FA) = 90° was used. Tumor volumes were manually defined by drawing regions of interest (ROI) to estimate tumor tissue throughout the body area in each anatomical direction, and a 3D volume estimate was calculated. Three different ROIs were selected, and the average was estimated. The mean volume of the two-directional 3D estimate was presented as the total tumor volume^48^.

### Histological examination

The tumor tissues were extracted 7 days after IRE treatment. The harvested tumor tissues were fixed in 10% neutral buffered formalin for 24 h and embedded in paraffin. Samples were sectioned transversely for microscopic examinations. The slides were stained with hematoxylin and eosin (H&E) for histological analysis. Immunohistochemistry (IHC) was performed on paraffin-embedded sections using TUNEL (Apop Tag^®^ Peroxidase In Situ Apoptosis Detection Kit; Merck, Burlington, MA, USA) as the primary antibody to verify the occurrence of apoptosis after the IRE procedure and to measure the percentage of necroptosis and necrosis^46^. All scans of stained samples were performed using a digital slide scanner (Pannoramic 250 FLASH III; 3D HISTECH Ltd., Budapest, Hungary). Measurements were obtained using a digital microscopy viewer (CaseViewer, 3D HISTECH Ltd.). The analyses of the histological findings were based on the consensus of three observers blinded to group assignment.

### Immunofluorescence examination

Immunofluorescence (IF) staining was performed with the ROS1 (1:8,000 dilution; MA5-26760; Thermo Fisher Scientific) antibody. Secondary antibody staining was performed using Alexa Fluor 488 goat anti-mouse IgG (H + L) (1:400 dilution; A-11001; Invitrogen) and 4′-6-diamidino-2-phenylindole (DAPI; D9542; Sigma Aldrich). The number of positive-staining cells was subjectively determined according to the distribution and density of the cells. The average values for the number of positive-staining cells were calculated as 100 × (the number of positive cell/the number of total cells).

### Statistical analysis

Data are presented as mean ± standard deviation (SD). Statistical significance between the groups was determined using either a two-tailed paired Student’s t-test or one-way ANOVA (significance, *p < 0.05; **p < 0.01; ***p < 0.001), followed by Tukey’s post hoc analysis. Statistical analyses were performed using GraphPad Prism 8 software (GraphPad Software, San Diego, CA, USA) and SPSS (version 27.0; IBM Corp., Armonk, NY, USA).

### Ethics declarations

All experiments were performed in accordance with relevant ARRIVE guidelines and regulations. Approval for Animal Experiments All experiments were approved by the Institutional Animal Care and Use Committee of the Asan Institute for Life Sciences (IACUC-2020–13-349).

### Supplementary Information

Supplementary Information.

## Data Availability

The authors confirm that the data supporting the findings of this study are available within the article. Raw data that support the findings of this study are available from the corresponding authors upon reasonable request.
