# Supplementary material for: Effects of bipolar irreversible electroporation with different pulse durations in a prostate cancer mouse model
Source: Sci Rep. 2024 Apr 30;14:9902. doi: 10.1038/s41598-024-60413-y (PMC11061152; doi:10.1038/s41598-024-60413-y)
Supplement: Supplementary file 1 — Supplementary Information. [file 41598_2024_60413_MOESM1_ESM.docx]

**Effects of Bipolar Irreversible Electroporation with Different Pulse Durations in a Prostate Cancer Mouse Model**

*Song Hee Kim^1,2†^, Jeon Min Kang^1†^, Yubeen Park^1^, Yunlim Kim^3^, Bumjin Lim^3*^, Jung-Hoon Park^1,4*^*

^1^Biomedical Engineering Research Center, Asan Institute for Life Sciences, Asan Medical Center, 88 Olympic-ro 43-gil, Songpa-gu, Seoul, 05505, Republic of Korea

^2^Department of Gastroenterology, Asan Medical Center, University of Ulsan College of Medicine, 88 Olympic-ro 43-gil, Songpa-gu, Seoul, 05505, Republic of Korea

^3^Departments of Urology, Asan Medical Center, University of Ulsan College of Medicine, 88 Olympic-ro 43-gil, Songpa-gu, Seoul, 05505, Republic of Korea

^4^Department of Convergence Medicine, Asan Medical Center, University of Ulsan College of Medicine, 88, Olympic-ro 43-Gil, Songpa-gu, Seoul 05505, Republic of Korea

^†^S.H.K. and J.M.K. contributed equally to this work and are the co-first authors.

*B.L. and J.-H.P. contributed equally to this work and are the co-corresponding authors.

**Corresponding Author**

**Bumjin Lim M.D., Ph.D.**

Departments of Urology, Asan Medical Center, University of Ulsan College of Medicine, 88 Olympic-ro 43-gil, Songpa-gu, Seoul, 05505, Republic of Korea
Tel: 82-8-3010-1835, Fax: 82-2-476-0090
E-mail: [lbj1986@hanmail.net](mailto:lbj1986@hanmail.net)

**Jung-Hoon Park, Ph.D.**

Department of Convergence Medicine, Asan Medical Center, University of Ulsan College of Medicine, 88 Olympic-ro 43-gil, Songpa-gu, Seoul 05505, Republic of Korea

Biomedical Engineering Research Center, Asan Institute for Life Sciences, Asan Medical Center, 88 Olympic-ro 43-gil, Songpa-gu, Seoul 05505, Republic of Korea

Tel: 82-2-3010-4123 Fax: 82-2-476-0090
E-mail: [jhparkz@amc.seoul.kr](mailto:jhparkz@amc.seoul.kr)

***
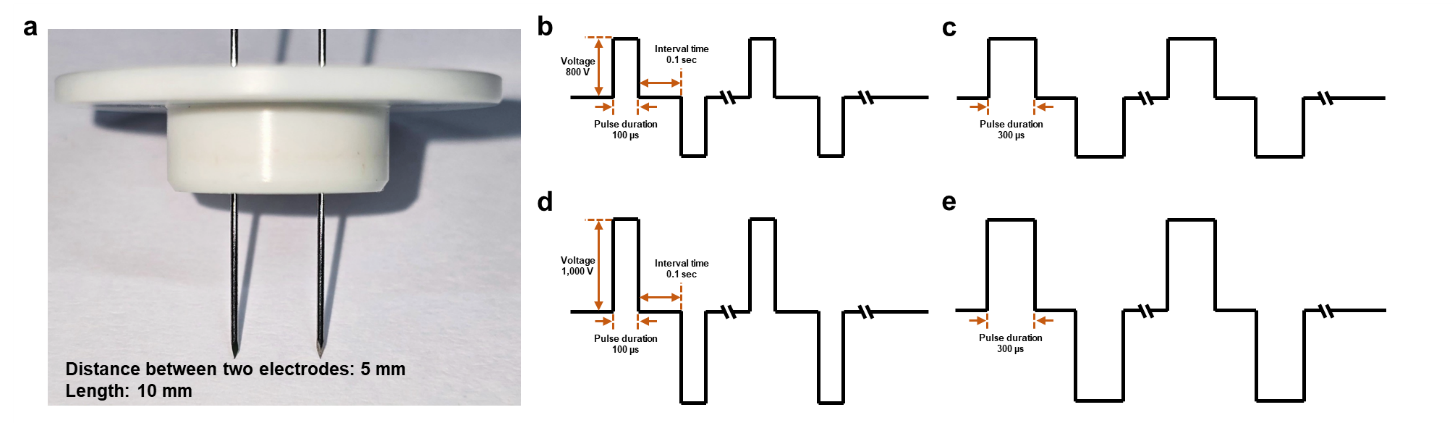
***

**Supplementary Figure 1.** Electrode and schematic of bipolar pulse configurations used in the study. (**a**) Photograph showing a 2-needle array electrode. IRE waveforms showing (**b**) 800 V and 100 μs of pulse duration, (**c**) 800 V and 300 μs of pulse duration, (**d**) 1,000 V and 100 μs of pulse duration, and (**e**) 1,000 V and 300 μs of pulse duration. Each waveform utilized an interval time of 0.1 sec, and a total of 40 pulses were applied.


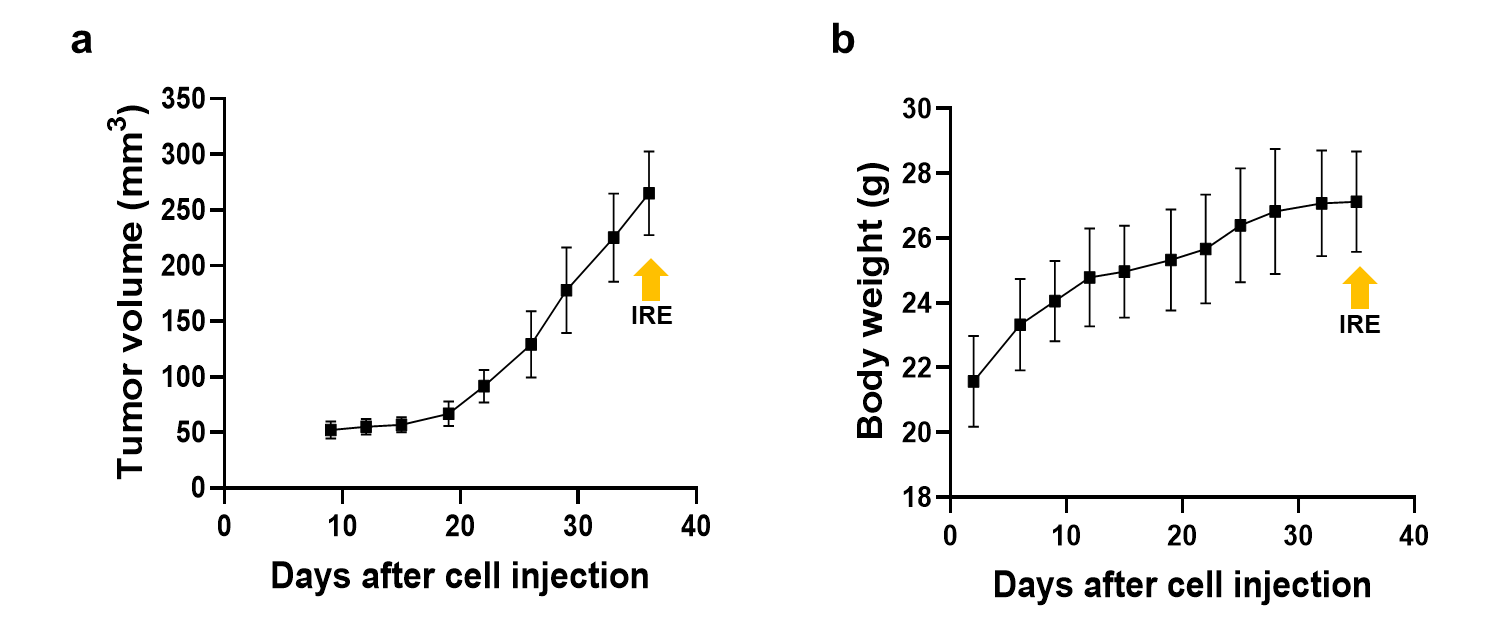


**Supplementary Figure 2.** *In vivo* tumor growth in the PC-3 models. (**a**) Average tumor volume growth curves following cell injection to just before IRE. (**b**) Average body weight change curves following cell injection to just before IRE treatment.

**Supplementary Table 1.** Tumor volume changes and histological findings of all study groups in IRE-treated prostate cancer mouse model

|  | **Groups** | | | | | ***p*-value** |
| --- | --- | --- | --- | --- | --- | --- |
|  | Sham control | 800 V  (100 μs) | 800 V  (300 μs) | 1,000 V  (100 μs) | 1,000 V  (300 μs) |  |
| Tumor volume at 7 days (mm^3^) | 355.98 ± 5.19 | 291.87 ± 5.13 | 289.65 ± 4.15 | 309.92 ± 4.32 | 280.23 ± 6.53 | < .001 |
| Tumor weight at 7 days (g) | 0.21 ± 0.01 | 0.14 ± 0.02 | 0.13 ± 0.02 | 0.19 ± 0.01 | 0.15 ± 0.01 | < .001 |
| Percentage of tumor volume (%) | 111.71 ± 1.27 | 62.75 ± 5.38 | 34.33 ± 3.30 | 48.13 ± 2.95 | 26.79 ± 3.54 | < .001 |
| Necroptosis (%) | 6.80 ± 3.56 | 22.54 ± 2.43 | 39.21 ± 3.87 | 31.69 ± 4.38 | 35.96 ± 3.38 | < .001 |
| Necrosis (%) | 0.62 ± 0.45 | 18.31 ± 2.47 | 39.78 ± 2.01 | 23.98 ± 4.68 | 52.33 ± 4.09 | < .001 |
| TUNEL-positive cells (Degree) | 1.17 ± 0.41 | 2.33 ± 0.52 | 4.17 ± 0.75 | 3.17 ± 0.41 | 4.50 ± 0.55 | < .001 |
| ROS1-positive cells (%) | 11.53 ± 1.29 | 29.57 ± 1.14 | 52.08 ± 1.17 | 39.37 ± 1.29 | 57.56 ± 1.84 | < .001 |

Note. Data are presented as means ± standard deviations

**Supplementary Table 2.** Comparison of tumor changes and histological results between study groups in IRE-treated prostate cancer mouse model

| ***p*-value** | | | | | | | | | | |
| --- | --- | --- | --- | --- | --- | --- | --- | --- | --- | --- |
|  | Sham control  vs.  800 V (100 μs) | Sham control  vs.  800 V (300 μs) | Sham control  vs.  1,000 V (100 μs) | Sham control  vs.  1,000 V (300 μs) | 800 V (100 μs) vs.  800 V (300 μs) | 800 V (100 μs)  vs.  1,000 V (100 μs) | 800 V (100 μs) vs.  1,000 V (300 μs) | 800 V (300 μs) vs.  1,000 V (100 μs) | 800 V (300 μs) vs.  1,000 V (300 μs) | 1,000 V (100 μs) vs.  1,000 V (300 μs) |
| Tumor volume (mm^3^) | < .001 | < .001 | < .001 | < .001 | .417 | < .001 | < .01 | < .001 | .225 | < .001 |
| Tumor weight (g) | < .001 | < .001 | .187 | < .001 | .956 | < .001 | .627 | < .001 | .250 | < .001 |
| Percentage of tumor volume (%) | < .001 | < .001 | < .001 | < .001 | < .001 | < .001 | < .001 | < .001 | < .01 | < .001 |
| Necroptosis (%) | < .001 | < .001 | < .001 | < .001 | < .001 | < .01 | < .001 | < .05 | .526 | .266 |
| Necrosis (%) | < .001 | < .001 | < .001 | < .001 | < .001 | < .05 | < .001 | < .001 | < .001 | < .001 |
| TUNEL-positive cells (Degree) | < .01 | < .001 | < .001 | < .001 | < .001 | .088 | < .001 | < .05 | .822 | < .01 |
| ROS1-positive cells (%) | < .001 | < .001 | < .001 | < .001 | < .001 | < .001 | < .001 | < .001 | < .001 | < .001 |
